# CT perfusion-guided thrombolysis in the extended window: from trial eligibility to individualized patient selection

**DOI:** 10.3389/fneur.2026.1928500

**Published:** 2026-09-15

**Authors:** Qing Hao, Dylan Ryan, Salman Ikramuddin

**Affiliations:** Department of Neurology, Duke University School of Medicine, Durham, NC, United States

**Keywords:** extended window, implemenation, stroke, thrombolysis (for acute ischaemic stroke), TNK

## Abstract

Recent randomized controlled trials (RCTs) have expanded the role of intravenous thrombolysis (IVT) into the extended time window (beyond 4.5 h) using CT perfusion (CTP)-based patient selection. Following the inclusion of extended-window thrombolysis into the most recent edition of the American Heart Association Acute Ischemic Stroke Guidelines in 2026, these recently completed studies, EXTEND, HOPE, TRACE III, and OPTION, collectively must now inform the rapid clinical implementation of this treatment as we enter an age of precision medicine in the management of acute stroke. This mini-review seeks to address three core implementation challenges for patient selection when considering extended-window thrombolysis: (1) efforts to establish clinical pathways for patient selection should consider the phenotype of patients enrolled in extended-window thrombolysis RCTs in addition to enrollment criteria, (2) assessments of the safety of extended-window thrombolysis based on the best available evidence are reassuring, and (3) promising results from extended-window thrombolysis trials for distal and medium vessel occlusion (DMeVO) stroke patients, in concert with recent neutral endovascular therapy (EVT) trials for treating DMeVO stroke, have carved out a distinct role for IVT in this patient population. Herein, we aim to leverage the best available evidence to address these critical questions that vascular neurologists face as we work toward the clinical implementation of this trial evidence.

## 1 Introduction

Recent trials, such as *Tenecteplase for Ischemic Stroke at 4.5 to 24 Hours without Thrombectomy* [TRACE III] (1), *Alteplase for Acute Ischemic Stroke at 4.5 to 24 Hours: The HOPE Randomized Clinical Trial* [HOPE] (2), *Thrombolysis Guided by Perfusion Imaging up to 9 Hours after Onset of Stroke* [EXTEND] (3), and now *Tenecteplase for Acute Non-Large Vessel Occlusion 4.5 to 24 Hours After Ischemic Stroke: The Option Randomized Clinical trial* [OPTION] (4), have materially advanced our understanding of how to medically revascularize patients in the extended window (4.5–24 h). These trials culminated in the publication of the 2026 Guidelines for the Early Management of Patients With Acute Ischemic Stroke, which assigned class 2a and 2b evidence for the use of CT perfusion (CTP)-guided Tenecteplase administration in patients who are 0–9 h and 0–24 h from their last known well state, respectively (5). These advances will improve clinical outcomes in stroke treatment, reduce long-term disability following stroke, and increase the number of patients eligible for revascularization therapy. However, before this vision can be fully realized, selecting patients for IVT using CTP in the extended window also presents considerable challenges. Herein, our mini-review aims to facilitate this transition in care pathways by emphasizing the need for rapid, individualized risk stratification and assessment of clinical benefits based on the best available evidence.

## 2 Evaluating the phenotype of patients likely to benefit from extended-window thrombolysis

As clinical protocols for extended-window thrombolysis are established, evidence-based practitioners will refer to the inclusion criteria of the relevant randomized clinical trials to inform and individualize patient selection in clinical settings. Understandably, patient selection for extended-window thrombolysis must begin with careful consideration of the inclusion criteria established in supportive randomized trials. EXTEND, HOPE, and OPTION all selected patients with National Institutes of Health Stroke Scale (NIHSS) scores of 4–25 or 4–26, and enrolled subjects with CTP core volumes <70 mL, penumbra-to-core ratio >1.2, and absolute mismatch of 10 mL. Imaging selection criteria have been summarized in Table 1. Despite these criteria, a review of these data suggests that the population of patients actually enrolled and treated successfully was a distinct phenotype. While enrollment criteria allow for the NIHSS score as high as 26, core volumes up to 70 cc, and mismatch ratios as low as 1.2 between 0–24 h, the median enrolled subject across these studies was more reflective of a patient with an NIHSS score ranging from 7 to 17, with target vessel occlusions in the M1 and M2 segments, core volumes under 30 cc, and penumbral volumes of 30–120 cc (Table 1). Patients, on average, had far smaller core and penumbral estimates than what was allowed in the enrollment criteria. Considering these factors may help guide patient selection and provide a stronger rationale for treatment decisions in the acute setting. We question whether unconditional treatment of any patient who numerically meets enrollment criteria for HOPE or EXTEND is an optimal strategy, as the benefit from IVT is not likely to be equally distributed across the whole spectrum of patients who happen to meet trial inclusion criteria. Patients with larger mismatches, small cores, and substantial penumbral estimates are the most likely to benefit from IVT between 4.5 and 24 h, and the effect size of the intervention must be taken into account along with other individualized factors such as baseline functional status, relative contraindications for IVT, and baseline stroke severity in crafting an approach to reperfusion in the acute setting. Additional trial data and high-quality, well-powered secondary analyses are needed to inform this decision-making in the long run.

**Table 1:** Comparison of inclusion criteria and the enrolled populations in extended-window thrombolysis trials.

Trial	Key variables	Inclusion criteria	Enrolled population
EXTEND 2019 (*n* = 113)	Age NIHSS Atrial fibrillation (%) Stroke onset to treatment (h) CTP penumbra (ml) CTP core (ml) CTP mismatch (ml) CTP ratio Triage vessel^**^	18+ 4–26 – 0–9 h^*^ – <70 >10 >1.2 Anterior circulation	73.7 ± 11.7^A^ 12 [8–17]^B^ 40.7 7.2 [6.2–8.1]^B^ 74.3 [40.1–134.0]^B^ 4.6 [0–23.2]^B^ ~69.7 ~16.1 39% M1, 22% M2
TRACE III 2024 (*n* = 264)	Age NIHSS Atrial fibrillation (%) LKW to randomization (h) CTP penumbra (ml) CTP core (ml) CTP mismatch (ml) CTP ratio Triage vessel	18+ 6–25 – 4.5–24 – <70 >15 >1.8 Anterior circulation	67 [58-75]^B^ 11 [7–15]^B^ 18.6 12.3 [8.5–16.4]^B^ 119.1 [79.8–117.2]^B^ 16.4 [5.7–28.4]^B^ ~102.7 ~7.2 32.8% M1, 39.7% M2
HOPE 2025 (*n* = 186)	Age NIHSS Atrial fibrillation (%) Stroke onset to randomization (h) CTP penumbra (ml) CTP core (ml) CTP mismatch (ml) CTP ratio Baseline ASPECT Triage vessel	18+ 4–26 – 4.5–24 – <70 >10 >1.2 – Any occlusion	72 [62–80]^B^ 10 [6–15]^B^ 19.4 6.8 [5.4–9.2]^B^ 49 [29–85]^B^ 12 [4–28]^B^ ~37 ~4.1 9 [8–10]^B^ 27% M1, 20% M2
OPTION 2026 (*n* = 282)	Age NIHSS Known cardio embolism (%) Stroke onset to randomization (h) CTP penumbra (ml) CTP core (ml) CTP Mismatch (ml) CTP ratio Triage vessel^***^	18+ 4–25 – 4.5–24 – <50 >10 >1.2 M2-4, ACA, PCA	69 [59–75]^B^ 7 [5–9]^B^ 23.8 12.4 [8.7–16.8]^B^ 34.8 [20.6–58.2]^B^ 0 [0–3.7]^B^ ~34.8 ~infinite 30.5% M2, 17% ACA

^*^Midpoint of sleep.

^**^A clear identification of a vessel occlusion was not required.

^***^Intracranial stenosis permitted.

LKW: last known well; NIHSS: National Institutes of Health Stroke Scale; CTP: computed tomography perfusion.

A: mean ± standard deviation (SD).

B: median [interquartile range (IQR)].

## 3 Individualized safety assessment in extended-window thrombolysis

Safety assessments will also be key during clinical implementation. In order to effectively personalize care, an understanding of the safety profile of extended-window thrombolysis is needed. Rates of symptomatic intracranial hemorrhage (sICH) ranged from 2.8% (OPTION) to 6.2% (EXTEND). In HOPE specifically, the odds ratio (OR) of sICH was 3.23 (0.28–6.19). These data remain similar to the intracranial hemorrhage risk reported in early trials of alteplase for ischemic stroke (6). This suggests that the safety profile in the extended window when selecting based on CTP could be similar to that of unselected patients in the conventional window. Nevertheless, careful interpretation of non-contrast CT head remains important; in *Safety and efficacy of Tenecteplase in patients with wake-up stroke assessed by non-contrast CT* (*TWIST): a multicentre, open-label, randomised controlled trial* (7), patients selected only with CT head [infarct <1/3 of the middle cerebral artery (MCA) territory], NIHSS ≥ 3, and unknown time of onset were treated with IVT. While patients in the treatment arm did not have a disability advantage at 3 months, the rate of sICH was comparable to placebo (2% vs. 1%, adjusted OR 2.17, 95% confidence interval (CI) 0.53–8.87, *p* = 0.28). Similarly, in *Alteplase for Posterior Circulation Ischemic Stroke at 4.5 to 24 Hours* [EXPECTS] (8), a randomized trial of alteplase for posterior circulation stroke in the extended window guided only by a posterior-circulation ASPECTS score of ≥7 and clinical symptoms of an ischemic stroke, sICH within 36 h after randomization occurred in just 2 patients (1.7%) in the alteplase group and 1 patient (0.9%) in the standard treatment group [unadjusted risk ratio, 1.98; 95% CI 0.18–21.56]. Collectively, these randomized data, while likely underpowered to detect a low baseline rate of sICH, suggest that treatment with IVT has an acceptable safety profile in this population with an Alberta Stroke Program Early CT Score (ASPECTS) of 8–10.

CTP studies obtained as a part of this clinical workflow also offer value for risk stratification; the hypoperfusion intensity ratio (HIR) may be predictive of hemorrhagic transformation following reperfusion therapy (9). Additional safety data that become available from real-world practice will be a valuable source of information. Overall, the best available evidence today suggests that in patients with presentation phenotypes seen in extended-window thrombolysis trials (smaller core volumes (<30cc), penumbral volumes 30–120cc, and ASPECTS ≥9), treatment with IVT may yield expected rates of sICH similar to those in patients treated in the conventional window.

## 4 Selection of patients for endovascular therapy (EVT) and/or IVT in the extended window: a personalized medicine approach

The expansion of IVT eligibility beyond 4.5 h has fundamentally altered acute stroke decision-making in patients with large, medium, and distal vessel occlusion stroke. While extended-window thrombolysis provides an additional reperfusion option, it also introduces uncertainty regarding the relative roles of IVT and EVT in patients with varying stroke severity and vessel occlusion locations. Consequently, treatment selection should no longer be viewed as a binary decision regarding thrombolysis alone, but rather as an individualized choice among available reperfusion strategies.

### 4.1 There is no clear added benefit of IVT in addition to EVT in the extended window

Although recent trials have established CT perfusion-guided IVT as an effective reperfusion strategy in the extended window, clinicians must now determine how these data should be integrated with established EVT pathways. The key question is not whether reperfusion should be pursued, but rather which reperfusion strategy (IVT alone, EVT alone, or combination therapy) is most appropriate for a given patient. *Tenecteplase for Stroke at 4.5 to 24 Hours with Perfusion-Imaging Selection* [TIMELESS] was a multicenter, placebo-controlled trial that randomized patients with large vessel occlusion (LVO) stroke to EVT vs. combination of EVT and IVT when selected by CTP. Data from TIMELESS suggested that while there was a similar rate of sICH in both groups, there was no improvement on the modified Rankin Scale (mRS) at 3 months when comparing EVT vs. IVT + EVT (10). Consequently, clinicians are increasingly faced with determining which patients are best served by EVT alone and which may derive greater benefit from IVT-based reperfusion strategies, particularly in clinical scenarios where the role of EVT remains uncertain. One important caveat remains: median dwell time for IVT in TIMELESS was just 16 min, a care benchmark difficult to replicate outside of the ideal conditions inside a comprehensive stroke center (CSC); there could be a role for combination therapy in patients with higher anticipated dwell time, such as those undergoing transfer to a CSC (11). In modern clinical practice, the vast majority of patients presenting with proximal large vessel occlusions and NIHSS ≥6 will undergo EVT between 0–24 h based on established clinical pathways. Given the importance of selecting the appropriate reperfusion strategy in the context of this emerging literature, we will now evaluate three additional key clinical scenarios involving IVT to aid in building a coherent treatment schema: (1) patients with mild stroke severity (NIHSS ≤5), (2) those with dominant medium vessel occlusion stroke, and (3) those with non-dominant medium vessel occlusion stroke.

### 4.2 Low NIHSS vessel occlusion stroke

Patients presenting with low NIHSS scores represent one of the most challenging populations in acute stroke care. Although many have large or medium vessel occlusions with salvageable brain tissue, their relatively mild presenting deficits reduce the expected absolute benefit of reperfusion therapy while preserving the potential risks of thrombolysis and endovascular intervention. Determining which patients with low NIHSS are most likely to benefit from treatment, therefore, remains an important area of ongoing investigation. *In Tenecteplase versus standard of care for minor ischaemic stroke with proven occlusion (TEMPO-II): a randomised, open-label, phase 3 superiority trial* (12), investigators randomized patients with identified vessel occlusion stroke, minor symptoms (NIHSS 0–5), and a perfusion abnormality between 0–12 h from stroke onset to Tenecteplase vs. placebo. TEMPO-II demonstrated that the use of IVT did not provide a disability benefit in patients with low NIHSS and non-disabling symptoms. Of the extended-window thrombolysis trials, HOPE and OPTION included patients with NIHSS ≥4, with their first-quartile NIHSS values being 6 and 5, respectively. Collectively, HOPE and OPTION had approximately a quarter of patients with NIHSS scores of approximately 4–6. Given this, clinicians making individualized reperfusion decisions must consider whether stroke symptoms are disabling (as they were in HOPE) or non-disabling (as they were in TEMPO-II). IVT in the population of patients with disabling stroke and NIHSS 4–6 could be reasonable in the extended window when guided by CTP. *Endovascular Therapy for low NIHSS Ischemic Stroke* [ENDOLOW] (13) is an ongoing trial that will provide data on the use of EVT in patients with NIHSS ≤5.

### 4.3 Extended window IVT for dominant medium vessel occlusion stroke

Among medium vessel occlusions, dominant M2 lesions present one of the greatest opportunities for individualized treatment selection. Their variable vascular anatomy, heterogeneous clinical presentation, and limited randomized evidence beyond 6 h necessitate careful integration of vessel anatomy, stroke severity, and CT perfusion findings when selecting the optimal reperfusion strategy. Three landmark trials in 2025 tested EVT for distal and medium vessel occlusion stroke, finding no disability benefit at 3 months, even in higher NIHSS subgroups (14). The 2026 Guidelines for the Early Management of Patients With Acute Ischemic Stroke, therefore, outlined class 2a evidence supporting EVT only in *dominant* M2 occlusions between 0 and 6 h from last known well (5). In clinical practice, this makes the determination of dominant and non-dominant M2 occlusions critical. The wide anatomic variations seen in M2 branching anatomy present a challenge to the vascular and interventional neurologist. In the most common anatomy, when comparing the inferior and superior division M2, the division that supplies the majority of the parietal lobe is the dominant M2 (15). There remains limited evidence for the treatment of dominant M2s beyond 6 h, regardless of stroke severity. At the current juncture, this population may be best suited for treatment with CTP-guided IVT between 6 and 24 h. Importantly, the extended-window thrombolysis trials included many patients with M2 occlusions, with enrollment rates ranging from 20% in HOPE to as high as ~40% in TRACE III.

### 4.4 Extended-window IVT for distal and non-dominant medium vessel occlusion stroke

Among all ischemic stroke subgroups, distal and non-dominant medium vessel occlusions remain among the least well defined with respect to reperfusion therapy. The absence of consistent randomized evidence supporting EVT, coupled with recent data supporting CT perfusion-guided IVT, has created a therapeutic gray zone in which treatment decisions must be individualized according to stroke severity, vascular anatomy, and imaging findings. *Endovascular Treatment of Medium-Vessel-Occlusion* Strokes [ORIENTAL-MeVO] was an open-label randomized trial which was recently presented at the International Stroke Conference. ORIENTAL-MeVO allocated patients with *non-dominant* M2, M3, A1-3, and P1-P3 segment occlusions with NIHSS ≥6, 6 h from symptom onset to EVT vs. medical management if selected by clinical-imaging or perfusion imaging mismatch (mismatch volume ≥10 cc, ratio >1.4). The study demonstrated an adjusted odds ratio of 1.24 for functional independence (mRS 0–2) and 1.47 for excellent functional outcome (mRS 0–1) at 90 days (16). These subjects had a median NIHSS of 10, potentially supporting the use of EVT in DMeVO patients with higher stroke severity. However, a comparison to IVT-based reperfusion outcomes is needed to inform a treatment decision in everyday practice. To facilitate this comparison, we have compiled an appraisal of the ORIENTAL-MeVO, HOPE, and OPTION trials (Table 2), which enrolled patients with a similar stroke severity (NIHSS 10 in HOPE) and target vessel occlusion (DMeVO in OPTION), respectively. In these trials, the adjusted odds ratio for functional independence (mRS 0–2) in favor of the treatment groups was comparable. This creates considerable uncertainty regarding the choice of reperfusion therapy in the extended window, and more randomized data are needed to delineate the ideal reperfusion modality for patients with non-dominant M2 or DMeVO stroke. While ORIENTAL-MeVO supports EVT in those with more severe stroke (NIHSS ~10), OPTION also demonstrated the benefit of IVT in this population. Additional randomized data are needed to inform this clinical decision, and existing clinical protocols must consider individual patient factors such as age, baseline functional impairment, procedural risk, anatomical considerations relevant to EVT, and relative contraindications to IVT.

**Table 2:** Comparison of representative extended-window IVT and EVT trials in non-dominant medium and distal vessel occlusion stroke.

Intervention	HOPE 2025 (*n* = 186) IVT	ORIENTAL-MeVO 2026 (*n* = 280) EVT	OPTION 2026 (*n* = 282) IVT
Baseline characteristics			
Age	72 [62–80]	71 [62–77]	69 [59–75]
NIHSS	10 [6–15]	10 [8–16]	7 [5–9]
Stroke onset to randomization (h)	6.8 [5.4–9.2]	5.0 [3.4–8.2]	12.4 [8.7–16.8]
Occlusion site			
M1	27.4%	–	–
M2	19.4%	43.9%	30.5%
M3-4	20.4%	13.2%	8.5%
ACA	4.8%	23.6%	17.0%
PCA	2.2%	19.3%	13.1%
IVT (%)	100%	36.1%	100%
Clinical outcomes			
mRS 0–2 (aOR, 95% CI)	1.20 (1.00–1.45)^NS^	1.24 (1.24, 1.07–1.44)	1.16 (1.02–1.32)
mRS 0–1 (aOR, 95% CI)	1.52 (1.14–2.02)	1.47 (1.20–1.78)	1.32 (1.08–1.61)
sICH (aOR, 95% CI) sICH (%)	3.23 (0.28–6.19) 3.8%	2.21 (0.87–5.62)^NS^ 4.7%	– 2.8%

Continuous variables are presented as median (interquartile range [IQR]).

NS, non-significant; IVT, intravenous thrombolysis; EVT, endovascular therapy; NIHSS, National Institutes of Health Stroke Scale; ACA, anterior cerebral artery; PCA, posterior cerebral artery; mRS, modified Rankin Scale; aOR, adjusted odds ratio; CI, confidence interval; sICH, symptomatic intracranial hemorrhage.

Representative randomized trials were selected because they enrolled patients with comparable stroke severity and medium/distal vessel occlusion anatomy, facilitating qualitative comparison of reperfusion strategies.

## 5 Conclusion

Recent trials support the safe and effective use of CTP-guided IVT in the extended window, but careful attention to the phenotype of enrolled patients, rather than simply the inclusion criteria, is essential for real-world implementation. Patients with moderate stroke severity, small core volumes, and substantial penumbral tissue appear more likely to benefit, with hemorrhagic risk comparable to conventional window treatment. There remains uncertainty regarding the optimal role of EVT in patients with DMeVO and higher stroke severity, underscoring the need for nuanced and individualized decision-making as well as high-quality randomized evidence to refine clinical practice in the coming years. Broader implementation of extended-window thrombolysis protocols remains limited by the nationwide availability of CTP.
